# Supplementary material for: Factors involved in vulvar pain during sexual activity and persistence in sexual activity amidst pain
Source: PLoS One. 2025 May 29;20(5):e0306086. doi: 10.1371/journal.pone.0306086 (PMC12122030; doi:10.1371/journal.pone.0306086)
Supplement: S1 File — (DOCX) [file pone.0306086.s001.docx]

**Supporting information**

**S1 File. Complete questionnaire.**
