# Supplementary material for: Factors involved in vulvar pain during sexual activity and persistence in sexual activity amidst pain
Source: PLoS One. 2025 May 29;20(5):e0306086. doi: 10.1371/journal.pone.0306086 (PMC12122030; doi:10.1371/journal.pone.0306086)
Supplement: S2 File — (DOCX) [file pone.0306086.s002.docx]

**Supporting information**

**S2 File. Confirmatory Factor Analysis.**

**Sexual Agency.** To generate a measure for sexual agency, a principal component analysis of all items of the self-constructed “Definition of sex and sexual agency Questionnaire” related to the concept of sexual agency (defined as “the ability to identify, communicate, and negotiate one's sexual needs, and to initiate behaviors that allow for the satisfaction of those needs”) was performed. Initially, 11 items were entered, including *provide pleasure* and *meet expectation* scores, items measuring initiation of sexual activity, faking orgasms, engaging in undesired sexual activity, saying no to undesired sex, and feeling guilty or embarrassed for – and taking time in engaging in sexual play that is not specifically stimulating for one’s partner. A varimax (orthogonal) rotation was used, maximizing the variance of squared loadings within each factor, and yielding factors that are uncorrelated to each other. Based on the Bartley’s test of Sphericity, the correlations among the variables were statistically different to an identity matrix ($\chi^{2}$ (55) = 563.67, p < .001), indicating that the data provides a meaningful structure to be explored via PCA. Four components with Eigenvalues above 1 (Kaiser, 1960) were identified, corresponding to the Scree Plot (Catell, 1966; see Graphic 1). Based on the given communalities and the rotated component matrix, the item measuring *faking orgasms* could be removed, while the remaining items (n = 10) each load strongly on one of the four factors (see Table 1). The four factors cumulatively explain 64 % of the variance explained by the original variables. Factors 1 and 2 explain a cumulative variance of 42 %.

**Graphic 1**

*Scree Plot yielded by Confirmatory Factor Analysis*


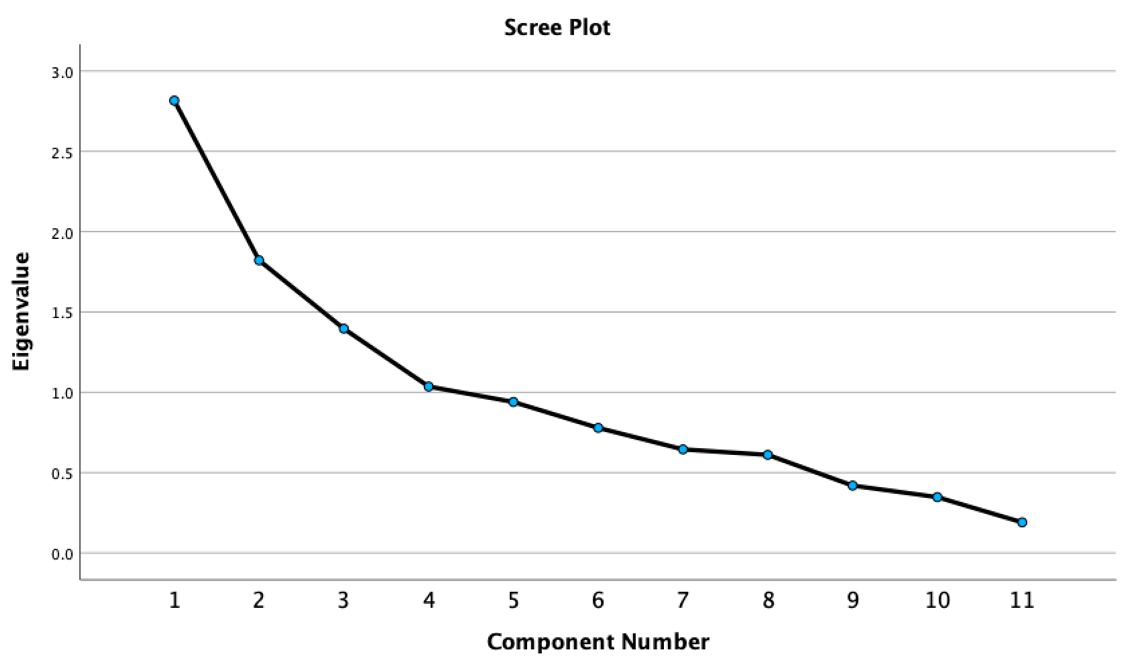


| **Table 1**  *Communalities and Rotated Component Matrix* | | | | | |
| --- | --- | --- | --- | --- | --- |
| **Item** | **Communalities**  Extraction | **Factor 1** | **Factor 2** | **Factor 3** | **Factor 4** |
| I prefer my partner to initiate sexual activity | .651 | .041 | -.067 | .072 | **.800** |
| I prefer to initiate sexual activity myself | .553 | .028 | .063 | .393 | **.627** |
| Meet Expectation | .858 | -.063 | **.922** | .056 | .028 |
| Provide Pleasure | .852 | -.081 | **.917** | .068 | -.029 |
| ~~I fake orgasms~~ | ~~.362~~ | ~~.323~~ | ~~-.361~~ | ~~-.245~~ | ~~.259~~ |
| I am afraid to say no to sex | .725 | **.804** | -.120 | .192 | -.168 |
| I engage in sexual activity I do not desire | .664 | **.656** | -.338 | .286 | -.194 |
| I feel guilty about taking time in sexual play which may not be specifically stimulating to my partner | .647 | **.769** | .004 | -.190 | .139 |
| I feel embarrassed asking for stimulation that does not specifically stimulate my partner | .706 | **.770** | .004 | -.167 | .292 |
| I actively seek/initiate sexual activity with my partner | .598 | .183 | .172 | **.722** | .120 |
| I take time in sexual play which may not be specifically stimulating to my partner | .453 | .186 | .011 | **-.630** | -.144 |

Factors 1 and 2 explain a cumulative variance of 42%.

**Factor 1.**

Subscale 1 is comprised of the items *I am afraid to say no to sex, I engage in sexual activity I do not desire, I feel guilty about taking time in sexual play which may not be specifically stimulating to my partner,* and *I feel embarrassed asking for stimulation that does not specifically stimulate my partner*, which load strongly on Factor 1. Subscale 1 is named **Shame & Engagement in undesired sexual activity** and yields an internal consistency score of $\alpha$ = .76.

**Factor 2.**
 The items *I engage in the following behaviours****solely to provide pleasure to my partner* (provide pleasure) and** *I engage in the following behaviours****solely to meet my partner's expectation* (meet expectation)** load strongly on Factor 2 and comprise Subscale 2, named **Partner’s pleasure prioritization**. Subscale 2 yields an internal consistency score of $\alpha$ = .90. The subscales are coded on a 5-point Likert scale from (0) to (4), in which high scores indicate high sexual agency, i.e., low partner’s pleasure prioritization and low shame and engagement in undesired sexual activity.
