# Supplementary material for: Factors involved in vulvar pain during sexual activity and persistence in sexual activity amidst pain
Source: PLoS One. 2025 May 29;20(5):e0306086. doi: 10.1371/journal.pone.0306086 (PMC12122030; doi:10.1371/journal.pone.0306086)
Supplement: S1 Table — (DOCX) [file pone.0306086.s003.docx]

**Supporting information**

**S1 Table. Covariances and correlations of predictor variables.**

|  |  | PP Prio | Shame | SSEI | Definition | Motivation | |
| --- | --- | --- | --- | --- | --- | --- | --- |
| Partner Pleasure Prio  Shame  SSEI  Definition of Sex  Sexual Motivation | Covariance  Correlation  Covariance  Correlation  Covariance  Correlation  Covariance  Correlation  Covariance  Correlation | -  1  −.109*  -.205  −.398*  .149  .141*  .271  -.007  -.010 | −.109*  −.205  -  1  1.679*  .519  −.009  -.014  .132*  .151 | −.398*  .149  1.679*  .519  -  1  .057  .018  2.415*  .550 | .141*  .271  −.009  -.014  .057  .018  -  1  .100  .117 | | −.007  -.010  .132*  .151  2.415*  .550  .100  .117  -  1 |
