# Supplementary material for: Factors involved in vulvar pain during sexual activity and persistence in sexual activity amidst pain
Source: PLoS One. 2025 May 29;20(5):e0306086. doi: 10.1371/journal.pone.0306086 (PMC12122030; doi:10.1371/journal.pone.0306086)
Supplement: S2 Table — (DOCX) [file pone.0306086.s004.docx]

**S2 Table. Standardized and unstandardized coefficients of final model.**

|  |  | **ß** | **b** | **S.E.** | **P** |
| --- | --- | --- | --- | --- | --- |
| Sex Despite Pain | Restrictive Definition of Sex | -,021 | -,035 | ,108 | ,745 |
|  | Autonomous Sexual Motivation | -,253 | -,303 | ,091 | *** |
|  | Partner Pleasure Prio (SA1) | -,026 | -,050 | ,129 | ,696 |
|  | Shame (SA2) | ,250 | ,407 | ,121 | *** |
|  | SSEITotal | ,105 | ,034 | ,028 | ,227 |
|  |  |  |  |  |  |
| Pain Communication | Restrictive Definition of Sex | ,173 | ,104 | ,040 | ,009 |
|  | Autonomous Sexual Motivation | -,119 | -,052 | ,033 | ,122 |
|  | Partner Pleasure Prio (SA1) | -,157 | -,111 | ,047 | ,019 |
|  | Shame (SA2) | ,023 | ,014 | ,044 | ,758 |
|  | SSEITotal | -,167 | -,020 | ,010 | ,060 |
|  |  |  |  |  |  |
| Relationship Satisfaction | Restrictive Definition of Sex | -,108 | -,208 | ,115 | ,071 |
|  | Autonomous Sexual Motivation | -,056 | -,078 | ,097 | ,424 |
|  | Partner Pleasure Prio (SA1) | ,069 | ,157 | ,138 | ,254 |
|  | Shame (SA2) | -,075 | -,141 | ,129 | ,273 |
|  | SSEITotal | ,558 | ,209 | ,030 | *** |
| FSFI |  |  |  |  |  |
|  | Restrictive Definition of Sex | -,040 | -,430 | ,609 | ,480 |
|  | Autonomous Sexual Motivation | ,272 | 2,096 | ,519 | *** |
|  | Partner Pleasure Prio (SA1) | ,121 | 1,525 | ,725 | ,036 |
|  | Shame (SA2) | ,029 | ,306 | ,687 | ,656 |
|  | SSEITotal | ,142 | ,295 | ,174 | ,089 |
|  | Relationship Satisfaction | ,284 | 1,585 | ,341 | *** |
|  | Sex Despite Pain | ,089 | ,572 | ,365 | ,117 |
|  | Pain Communication | -,107 | -1,904 | ,992 | ,055 |
|  |  |  |  |  |  |
| FSDS | Restrictive Definition of Sex | ,117 | 1,605 | ,640 | ,012 |
|  | Autonomous Sexual Motivation | ,005 | ,054 | ,545 | ,921 |
|  | Partner Pleasure Prio (SA1) | -,139 | -2,257 | ,761 | ,003 |
|  | SSEITotal | -,640 | -1,710 | ,182 | *** |
|  | Relationship Satisfaction | -,104 | -,743 | ,358 | ,038 |
|  | Shame (SA2) | -,136 | -1,818 | ,721 | ,012 |
|  | Sex Despite Pain | -,032 | -,262 | ,383 | ,494 |
|  | Pain Communication | -,141 | -3,223 | 1,041 | ,002 |
|  |  |  |  |  |  |
| Vulvar Pain | Restrictive Definition of Sex | ,152 | ,156 | ,063 | ,013 |
|  | Autonomous Sexual Motivation | ,054 | ,040 | ,054 | ,457 |
|  | Partner Pleasure Prio (SA1) | -,033 | -,040 | ,075 | ,596 |
|  | Shame (SA2) | -,002 | -,002 | ,071 | ,979 |
|  | SSEITotal | -,353 | -,071 | ,018 | *** |
|  | Relationship Satisfaction | ,101 | ,054 | ,035 | ,123 |
|  | Sex Despite Pain | -,306 | -,189 | ,038 | *** |
|  | Pain Communication | -,233 | -,400 | ,102 | *** |
