# Supplementary material for: Factors involved in vulvar pain during sexual activity and persistence in sexual activity amidst pain
Source: PLoS One. 2025 May 29;20(5):e0306086. doi: 10.1371/journal.pone.0306086 (PMC12122030; doi:10.1371/journal.pone.0306086)
Supplement: Attachment — Submitted filename: vulvar pain plosone.doc [file pone.0306086.s005.doc]

Vulvar pain PlosOne

This study tries to evaluate which psycho-social factors may explain the link between the presence of genital pain and the willingness to have penetrative sexual intercourse in heterosexual women. The authors reported interesting data. First, the presence of a very high prevalence of genital pain (80%) in a general population. Yet this information should be taken in mind and deserves a broader reflection on the female sexuality. Then the authors found a significant association between sexual distress, vulvar pain, and sexual function, with some other psychosocial variables: sexual self-esteem, definition of sex, sexual distress, sexual agency ,autonomous sexual motivation. These relationships were mediated by relationship satisfaction, sexual pain communication, and engagement in sexual intercourse despite pain.

I really appreciate this work, which gives to the readers a broader picture of the reasons why women experiencing pain engage in penetrative sex . I think that this work can be very useful from clinical point of view. Here I listed some minor comments.

Introduction

If I correctly understand, this study is aiming at evaluating general painful sensations in relationship to sexual activity or intercourse. For this reason, I suggest adopting the name of “genital pain” rather than of “vulvar pain” (as adopted by the authors) through the manuscript.

Where the authors mention the types of vulvar pain, indicating the “idea of penetration”, I suggest adding the possible diagnosis of vaginismus.

The authors refer to PVI of heterosexual women. It could be also useful to add the information of a lack of data on non-heterosexual couples.

From my point of view, the introduction is too long. I suggest summarizing the central points related to the main topic.

Methods:

The authors have to explain how they structured the items evaluating vulvar pain and sexual intercourse.

It could be useful to add the information about the type of diagnosis related to genital pain and its prevalence. Another important variable is the type of treatment and its efficacy in acting on pain management.

Definition of sexual agency: I don’t understand if the authors adopted only a limited number of the items present in the Hite Report of Female Sexuality.

In the results, I suggest to better define the “restrictive definition of sex”.

I suggest reporting, in the text or with a table, the prevalence of sexual dysfunctions. I think that this data
