# Supplementary material for: Factors involved in vulvar pain during sexual activity and persistence in sexual activity amidst pain
Source: PLoS One. 2025 May 29;20(5):e0306086. doi: 10.1371/journal.pone.0306086 (PMC12122030; doi:10.1371/journal.pone.0306086)
Supplement: Attachment — Submitted filename: Rebuttal_Letter_PLOSone.docx [file pone.0306086.s007.docx]

Dear Dr. Marta Panzeri,

We would like to thank you, as well as the peer reviewers, for the time dedicated to our manuscript. We are delighted to read that the peer reviewers have recommended publication, and gladly accept your invitation to conduct the revision on our manuscript (PONE-D-24-21802), entitled "Factors involved in vulvar pain during sexual activity and persistence in sexual activity amidst painful experiences.". We highly appreciate the constructive and very helpful comments whilst we hope that the revised manuscript now fully qualifies for publication in *PLOS ONE.*

With warm regards,

Carlotta Oesterling, on behalf of the co-authors.

| **Responses to the reviewers** |  |
| --- | --- |
|  | *Thank you very much for the positive feedback and the appreciation of our work. We have found the detailed suggestions and textual recommendations very helpful, and thus we have implemented them meticulously, hoping that sections that were somewhat unclear are clearer now. Below, we have referred to each comment separately.* |
| ***Reviewer 1*** | |
| *1.* Place the young average age within the limits and must take this into account in the discussion | **Response:**  *Thank you for this suggestion. We have included the following section in the limitations:*  **In-text changes:**  **[Discussion, p. 29]** Further, the sample was, on average, very young. Including older women may be relevant to derive inferences on whether sexual agency increases, while engagement in unwanted or painful sexual behaviour decreases with age and whether the relevant psychosocial factors change across different periods in women’s life, for example before and after childbirth or post menopause. |
| *2.* Put the description of the self-constructed questionnaire in the tools section and not in the statistical analysis section | **Response:**  *In line with this observation, we have separated the section describing the procedure for deriving scores of the self-constructed questionnaire from the statistical analysis section.* |
| *3.* Always use the same number of decimals in percentages | **Response:**  *We appreciate the reviewer’s attention and have adjusted the decimals of all percentages in the manuscript for consistency.* |
| ***Reviewer 2*** | |
| This study tries to evaluate which psycho-social factors may explain the link between the presence of genital pain and the willingness to have penetrative sexual intercourse in heterosexual women. The authors reported interesting data. First, the presence of a very high prevalence of genital pain (80%) in a general population. Yet this information should be taken in mind and deserves a broader reflection on the female sexuality. Then the authors found a significant association between sexual distress, vulvar pain, and sexual function, with some other psychosocial variables: sexual self-esteem, definition of sex, sexual distress, sexual agency, autonomous sexual motivation. These relationships were mediated by relationship satisfaction, sexual pain communication, and engagement in sexual intercourse despite pain.  I really appreciate this work, which gives to the readers a broader picture of the reasons why women experiencing pain engage in penetrative sex. I think that this work can be very useful from clinical point of view. Here I listed some minor comments. | **Response:**  *Thank you very much for your comment and your appreciation of our work, as for the valuable feedback and suggestions.* |
| **Introduction** | |
| If I correctly understand, this study is aiming at evaluating general painful sensations in relationship to sexual activity or intercourse. For this reason, I suggest adopting the name of “genital pain” rather than of “vulvar pain” (as adopted by the authors) through the manuscript. | **Response:**  *We recognize that genital pain is a broader term and corresponds to the diagnosis of GPPPD. However, we have chosen to use the term 'vulvar pain' throughout this manuscript to reflect the specific anatomical focus of the study, which primarily examines pain localized to the vulvar and vestibular regions. We believe that maintaining specificity aligns more closely with the psychosocial and clinical variables assessed in this research, and hope that the reviewer can appreciate our reasoning and subsequent preference for vulvar pain over genital pain.* |
| Where the authors mention the types of vulvar pain, indicating the “idea of penetration”, I suggest adding the possible diagnosis of vaginismus. | **Response:**  *We agree with your suggestion and would like to clarify that consistent with your suggestion the diagnosis of vaginismus was included in the paragraph you have mentioned.*  **[Introduction, p. 3]**  “Pain symptoms persisting for over six months and causing significant sexual distress are classified as Genito-Pelvic Pain/Penetration Disorder [2], which includes vulvar pain during penetrative intercourse caused by involuntary contraction of the pelvic floor muscles upon attempted vaginal entry (vaginismus), pain (dyspareunia) localized to the vestibule of the vagina (provoked vestibulodynia), or at other vulvovaginal locations (vulvodynia), and fear and anxiety elicited by attempted penetration [2], or by merely the idea of penetration [3].” |
| The authors refer to PVI of heterosexual women. It could be also useful to add the information of a lack of data on non-heterosexual couples. | **Response:**  *We have made in-text changes in line with this suggestion, defining GPPPD for pain during penetrative intercourse, not necessarily PVI. Then we have proceeded to mention, as suggested, that the corresponding research is yet to be conducted in women sleeping with women due to a current lack of data on non-heterosexual couples.*  *When describing the aim of the study, we replaced PVI with sexual intercourse, as our research was not limited to women engaging in heterosexual sex (however, the sample size of women sleeping with women was too low to make reliable inferences on differences between the two groups). Throughout the document, we attempted to change PVI to penetrative intercourse where possible (i.e., when it is not exclusively referred to penile-vaginal intercourse in heterosexual couples). Lastly, we added to the* ***future directions*** *section that sampling women who engage in sexual intercourse with women may be of particular importance.*  **In-text changes:**  **[Introduction, p. 3]**  “Pain symptoms persisting for over six months and causing significant sexual distress are classified as Genito-Pelvic Pain/Penetration Disorder [2], which includes vulvar pain during penetrative intercourse caused by involuntary contraction of the pelvic floor muscles upon attempted vaginal entry (vaginismus), pain (dyspareunia) localized to the vestibule of the vagina (provoked vestibulodynia), or at other vulvovaginal locations (vulvodynia), and fear and anxiety elicited by attempted penetration [2], or by merely the idea of penetration [3].”  **[Introduction, p. 3]**  Experiencing vulvar pain during penetration can come with significant sexual distress and may negatively impact sexual arousal, desire, and lubrication. In light thereof, it is striking that research indicated that a substantial proportion of heterosexual women affected by vulvar pain proceed to have penile-vaginal intercourse (PVI) [4, 5], with only 50% of women effectively communicating the experience of pain to their partner [6, 7]. A representative survey study has further found that 60% of affected women indicated experiencing the motivation to continue to engage in PVI despite pain [6]. While – given the reduced focus on penetration – it is likely that these numbers are lower in non-heterosexual female couples, corresponding statistics have not yet been established.  **[Introduction, p. 5]**  The consequential deep embedding of values may partially explain why PVI remains the epitome of “normal” heterosexual sex [16, 17]  **[Discussion, p. 29]**  Actively sampling women who engage in sexual activity with women may be of particular value, as there may be less pressure to engage in penetrative intercourse and the definition of sex is potentially wider and less scripted. |
| From my point of view, the introduction is too long. I suggest summarizing the central points related to the main topic. | **Response:**  *Following this comment, we shortened the introduction and restricted the text to the most central points related to each of the central constructs.* |
| **Methods** |  |
| The authors have to explain how they structured the items evaluating vulvar pain and sexual intercourse. | **Response:**  *The structure of the items evaluating vulvar pain can be found on page 12, ll. 8.*  *In addition, we now clarified that the “vulvar pain” variable included as outcome variable in the model alongside FSDS and FSFI score is the item measuring whether the subject experienced physical discomfort or pain when engaging in penetrative/vaginal intercourse with one’s partner, and that the last-mentioned variable (whether it was engaged in intercourse despite pain) was included as mediating variable.*  *Other aspects of (the definition of) sexual intercourse were measured as part of the definition of sex and sexual agency questionnaires.*  **In-text changes:**  **[Method, p. 11]**  Four self-constructed items were made available to assess vulvar pain and engagement of sexual intercourse despite pain. The first item appraised whether physical discomfort or pain is normally experienced upon (attempted) penetration, which could be responded to on a 5-point Likert scale ranging from 0 (No) to 4 (Yes, always). The achieved score was included as outcome variable in the model alongside FSFI and FSDS score. Next, participants could choose one or more possible options for why the sexual intercourse could have been hurtful. Presented options were based on the Hite Report of Female Sexuality [47] and included e.g., limited arousal and lubrication, yeast infection, and an open response box to add unmentioned causes of pain. Further, participants were asked to rate their average pain during intercourse on a scale from (1) *no pain* to (10) *worst pain* as recommended for clinical assessments of pain intensity [48]. Lastly, on a scale from 0 (No) to 4 (Yes, always), participants indicated whether they engage in intercourse despite pain, which was included as mediating variable into the model. |
| It could be useful to add the information about the type of diagnosis related to genital pain and its prevalence. Another important variable is the type of treatment and its efficacy in acting on pain management. | **Response:**  *This study relied on an unselected student sample and did not involve a formal clinical assessment interview, nor did we ask about their treatment status. However, findings from the pertinent measures did show that 51.2% of women scored above the cut-off score of 11 of the FSDS, indicating significant sexual distress and 48.3% of women scored below the cut-off score of 26, indicating female sexual dysfunction. This had been presented in the result section. Additionally, we now included the cut-off scores as defined by existing literature to the method of our manuscript.*  **In text changes:**  **[Method, p. 13]**  A cutoff score of < 26 indicates the presence of clinical sexual dysfunction [52].  **[Method, p. 14]**  A cut-off score of ≥ 15 was validated to indicate the presence of sexually related distress in a female sample [53]. |
| Definition of sexual agency: I don’t understand if the authors adopted only a limited number of the items present in the Hite Report of Female Sexuality. | **Response:**  *Only a limited number of items were adapted for the sexual agency questionnaire. Specifically, the coded responses to some questions published in the Hite report served as guidance for the self-constructed questionnaire and the response options provided. We could see that this was not sufficiently clear and have now clarified that only a limited number of items were adapted or served as guidance.*  **In text changes:**  **[Method, p. 15]**  **The definition of sex and sexual agency**  To assess remaining psychosocial aspects that are not captured by the herein administered validated questionnaires (i.e., the definition of sex and sexual agency**)**, a self-constructed questionnaire based on a number of questions presented in the Hite Report of Female Sexuality [47, 56] was utilized. Coded qualitative responses published in the report were used as guidance for the presentation of response options. |
| **Results** | |
| In the results, I suggest to better define the “restrictive definition of sex”. | **Response:**  *To more clearly define the term “restrictive definition of sex”, we have now included a definition into the result section: “Holding a definition of sex limited to penetrative sexual intercourse.”*  *A detailed description of how we arrived at a score reflecting the level to which participants displayed a restrictive definition of sex is presented in the data reduction and statistical analysis section. In addition, we have now included a definition into the result section.*  **In text changes:**  **[Results, p. 24]**  A restricted definition of sex (i.e., holding a definition of sex limited to penetrative sexual intercourse), was associated with increased sexual distress whereas sexual self-esteem, relationship satisfaction, pain communication and both dimensions of sexual agency were associated with reduced sexual distress.  **[Data reduction, p. 12]**  To gain insights into the sexual scripts and the broadness of the definition of sexual activity, a sum score and the mean for *PVI with* and *without orgasm* and for *all behaviours excluding PVI with and without orgasm* (oral sex, anal sex, kissing, masturbation, mutual masturbation, and intimacy and physical touch without penetration) were calculated for the participants and their partners. In a next step, a difference score was calculated between the agreement to PVI with and without orgasm as being part of the definition of sex and other behaviours as being defined as “having sex” for participants (*definition of sex PVI – definition of sex no PVI*), building a score named the **definition of sex**, of which higher scores indicate a definition of sex that is more restricted to PVI related activities. |
| I suggest reporting, in the text or with a table, the prevalence of sexual dysfunctions. I think that this data could give important perspective on how painful conditions worsen sexual wellbeing. | **Response:**  *To address this request, we have listed the prevalence of impaired sexual functioning or pain within the present sample. Since the current study did not involve a formal diagnostic assessment it was not possible to provide more detailed information about the prevalence of particular sexual dysfuntions.*  **[Results, p. 19]**  The present sample had a mean FSDS score of *M* = 13.8 (*SD* = 11.25), with 51.2% of women scoring above the cut-off score of 11, indicating sexual distress. The mean FSFI score was *M* = 23.76 (*SD* = 8.9), with 48.3% of women below the cutoff score of 26, indicating female sexual dysfunction. For indications of orgasm frequency, see Table 1.  When asked about the presence of physical discomfort or pain during attempted or engagement in PVI with their partners, 75.9% of women reported experiencing pain *at least sometimes*. Furthermore, 15.7% of women indicated to experience pain *more than half of the time* during (attempted) PVI (see Table 2). Factors contributing to pain included extended or frequent sexual activity (29.7%), insufficient arousal or lubrication (51.3%), deep thrusts (23.7%), a partner with a too-large penis (17.2%), discomfort at the beginning of intercourse (16.4%), and yeast infections (8.2%). Additional factors given by participants included body tension, vaginismus, dyspareunia, presence of an intrauterine device, sexual trauma, and stress. The distribution of average pain level ratings in percentages is illustrated in Fig 2. |
