# Supplementary material for: Factors involved in vulvar pain during sexual activity and persistence in sexual activity amidst pain
Source: PLoS One. 2025 May 29;20(5):e0306086. doi: 10.1371/journal.pone.0306086 (PMC12122030; doi:10.1371/journal.pone.0306086)
Supplement: Attachment — Submitted filename: Rebuttal Letter Oesterling et al., 28.03.2025.docx [file pone.0306086.s008.docx]

| **Reviewer 2** | **Response to Reviewer** |
| --- | --- |
| How the authors explain the very high percentage of vulvar pain in their sample, compared with the literature data? Have the authors considered to go in depth with the analysis of the prevalence, considering separate sub-groups pf women? I think that sexual experience may profoundly change in 18- or 30-years women. | The sample consisted of university students with a mean age of *M* = 20.04 (*SD* = 2.06), and in light of the low number of women aged 23 and above (see statistic below), we did not make subgroups. We agree that it would be important to compare the prevalence to different age-groups in future studies with more heterogenous samples, as the highest prevalence of vulvar pain is found in women under 25. Further, the high percentage (75.9%) includes women who experience pain at least sometimes, and does not include sexual distress measures, which would be required for diagnosis of sexual dysfunction (DSM-5; ICD-11) and would lead to lower prevalence rates. Women indicating to experience pain at least half of the time represented 15.7% of the participants in the current study, which corresponds to other findings in the literature (Briken et al., 2020; Carter et al., 2019). For example, the 12-month prevalence of women experiencing a sexual pain in a representative survey study in Germany was found to be 16.2% in women aged 18-25 (Briken et al., 2020). The 12-month prevalence of sexual pain accompanied with significant sexual distress is 9.4% (Briken et al., 2020). The study we replicated (Carter et al., 2019) found 23.3% of women to have experienced their last intercourse as painful – this statistic may be lower given the restriction to the last intercourse, while our question was phrased more generally.  Based on your comment, we have adjusted the corresponding limitations section to make this point more explicit.    In Text (p. 28)  “Further, the sample was, on average, very young, *which may have resulted in an increased prevalence of vulvar pain compared to older women (Briken et al., 2020).* *As prevalence of vulvar pain seems to decrease with age*, including older women may be relevant to derive inferences on whether sexual agency increases, while engagement in unwanted or painful sexual behaviour decreases with age and whether the relevant psychosocial factors change across different periods in women’s life, for example before and after childbirth or post menopause.” |
| The authors must explain why they decided to measure sexual motives adopting the construct of sexual scripts. | We have measured sexual motives using the sexual motives scale (sexMS), which is unrelated to sexual scripts. This is described in the method section on sexual motivation; therefore we hope that this is now clearer:  Sexual motivation was assessed using the sexual motives scale (SexMS) [43], a self-report questionnaire based on self-determination theory [42]. The SexMS consists of 24 items, with 4 items measuring each of six motivation styles: Amotivation, extrinsic motivation, introjected motivation, identified motivation, integrated motivation, and intrinsic motivation. Internal consistency showed to be good, with Cronbach’s α above .80 on all subscales. |
| The part dedicated to the description of sexual scripts should be shortened and more centered on the main topic of the study. | In order to keep the text more focused on the main topic of the study, in line with the reviewer’s recommendation we have shortened the respective section, removing and shortening sections on sexual scripts and sexual scripting theory. Therefore, the following paragraph on the restrictive definition of sex takes up more space as this is the more central aspect of the study, SST was just mentioned to give a sociocultural and historical background. |
| I would like to know if the authors have assesses in the first part of the study the presence of diagnosed sexual dysfunctions, of organic conditions suggesting the presence of pain, or of psychopathological traits/psychiatric diagnoses associated with vulvar pain. | As our study served the preliminary establishment of our model within a student sample and the inclusion criteria included sexually active women with no psychiatric diagnosis, we did not assess the proposed conditions in the first part of the study. However, we acknowledge that it is interesting and relevant to dive deeper into effects in women with chronic health conditions such as mental health conditions, cardiovascular and metabolic conditions, inflammatory and autoimmune conditions, chronic pain, and post-cancer (treatment). In future studies, the model is to be validated for women affected by chronic health conditions.  In the present paper, we clarified the inclusion criteria and added your suggestion to the limitation section.  “Sexually active women over 18 years of age were included into the sample, which consisted of *N* = 277 women between 18 and 33. Exclusion criteria comprised being pregnant, and being diagnosed with a psychiatric condition.”  “Lastly, SEM was used within the context of an inductive and data-driven approach to develop the model of best fit to represent the data. It is therefore important for future research to test the robustness of the final model in an independent sample. Future samples could extend to women affected by chronic health conditions such as mental health conditions, autoimmune or inflammatory diseases, chronic pain, or post-cancer (treatment). Women with sexual dysfunction or with partner’s diagnosed with sexual dysfunction could also be included to gain insights into the role of sexual agency and sexual communication in the development of secondary sexual dysfunction.” |
| I think that an assessment of partner’s sexual health could be important to detect. | We agree with the reviewer’s comment. While we assessed the partner’s definition of sex, the partner’s sexual health was not assessed. This is now added to the previous point in the limitation section (see in text change in response to previous comment). |
| The enrollment of an exclusive population of young women should represent a selection bias. I suggest adding this point to the limits. | Utilizing a homogenous sample comprised a necessary first step in establishing a clear and replicable foundation for our hypothesized model. As vulvar pain seems to be most prevalent in young women (Briken et al., 2020), limiting the first study of the model to female university students was a valuable starting point in order to test our model in a well-defined group. However, we acknowledge that from here on it is valuable to test the model in more heterogenous samples and have added the risk of selection bias pointed out by the reviewer to the limitation section.  “This study comprised a homogenous sample of female university students, of which the majority was heterosexual or indicated to engage in sexual activities with men and had a western and educated background. *While young women comprised a valuable starting point to text the hypothesized model, the homogenous sample renders the study vulnerable to selection bias. In a next step, it would therefore be important for future research to test whether the model generalizes to larger, more diverse samples*.” |
| I don’t understand if vulvar pain assessment is made with no-validated items. If yes, the authors are invited to explain the methodology for selecting and structuring these questions. | The vulvar pain items were self-constructed to optimally reflect the content of interest (see below for details). In addition, we included the FSFI (Rosen et al., 2000), a validated assessment tool for female sexual function in which pain is also assessed. Response options of the self-constructed items were based on coded responses of the Hite Report of Female Sexuality (Hite, 2003). Please see the respective part of the method section for more detailed information:  “Four self-constructed items were made available to assess vulvar pain and engagement of sexual intercourse despite pain. The first item appraised whether physical discomfort or pain is normally experienced upon (attempted) penetration, which could be responded to on a 5- point Likert scale ranging from 0 (No) to 4 (Yes, always). The achieved score was included as outcome variable in the model alongside FSFI and FSDS score. Second, participants could choose one or more possible options for why the sexual intercourse could have been hurtful. Presented options were based on the Hite Report of Female Sexuality [47] and included options such as limited arousal and lubrication, yeast infection, and an open response box to add unmentioned causes of pain. Third, participants were asked to rate their average pain during intercourse on a scale from (1) no pain to (10) worst pain as recommended for clinical assessments of pain intensity [48]. Fourth, on a scale from 0 (No) to 4 (Yes, always), participants indicated whether they engage in intercourse despite pain, which was included as mediating variable into the model.” |
| Pleasure evaluation: the authors propose to the subjects the following two questions:” I engage in the following behaviours solely to provide pleasure to my partner; I engage in the following behaviours solely to meet my partner's expectation.” From my point of view, an additional question matching together the two options (self- and partner- pleasure) is necessary | These questions were part of a larger set of questions on sexual agency that was later subject to factor analysis. Eleven items were entered into the analysis (see S2 file). The items measuring whether an individual engages in behaviours solely to meet the partner’s expectations and to provide pleasure to the partner were shown to be the only relevant items on the second sexual agency subscale.  Consistent with your point, items focused on the pursuit of own pleasure, for example: “I take time in sexual play which may not be specifically stimulating to my partner” were also included in the FA. However, these items did not significantly add to subscale two and are covered by the negatively phrased items included in subscale 2. For the rotated component matrix, see S2 file. |

References:

Briken P, Matthiesen S, Pietras L, et al. Estimating the prevalence of sexual dysfunction using the new ICD-11 guidelines. Dtsch Arztebl Int 2020;117(39):653-658. doi:10.3238/arztebl.2020.0653

Hite S. The Hite report: A nationwide study of female sexuality: Seven Stories Press; 2003.

Rosen R, Brown C, Heiman J, Leiblum S, Meston C, Shabsigh R, et al. The Female Sexual Function Index (FSFI): a multidimensional self-report instrument for the assessment of female sexual function. J Sex Marital Ther. 2000;26(2):191-208. doi: 10.1080/009262300278597.
